# Relationship Between Asthma and Headache Attacks in the Qassim Region, Saudi Arabia

**DOI:** 10.7759/cureus.39784

**Published:** 2023-05-31

**Authors:** Sami Alrasheedi, Kadi A Alhumaidi, Aeshah M Alharbi, Noura A Aldhowyan, Njood M Alobaid, Norah A Alturaif, Ghadi A Almatroudi, Moayed S Alkhalifah, Abdullah A Alrasheedi, Mousa N Alrashdi, Ahmad Alkhdairi

**Affiliations:** 1 Department of Medicine, Unaizah College of Medicine and Medical Sciences, Qassim University, Unaizah, SAU; 2 College of Medicine, Unaizah College of Medicine and Medical Sciences, Qassim University, Unaizah, SAU; 3 Department of Medicine, King Saud Hospital, Unaizah, SAU; 4 Department of Medicine, Buraidah Central Hospital, Buraidah, SAU

**Keywords:** asthma control, factors affecting headache, prevalence, asthma, headache

## Abstract

Background: Headaches are a common complaint among asthma patients. However, there is no study to assess the relationship between asthma and headaches or to assess the prevalence of headaches among asthma patients in Saudi Arabia. We aim to study the relationship between asthma and headaches and also to assess the prevalence of headaches among asthma patients.

Research methodology: We conducted a cross-sectional study among 528 asthmatic patients. Participants were selected through non-probability sampling from the system of four hospitals (King Fahad Specialist Hospital, King Saud Hospital, Buraidah Central Hospital, and Qassim University Hospital). The duration of our study was one year from 11 September 2022 to 14 May 2023. Data collection was performed by using a pre-tested and self-administered questionnaire. Data were analyzed through IBM SPSS Statistics for Windows, Version 24 (Released 2016; IBM Corp., Armonk, New York, United States) by using the chi-square test to assess the relationship between the qualitative variables and independent t-test and ANOVA for comparing the quantitative variables with a significant level set at p-value < 0.05.

Results: Five hundred and twenty-eight asthmatics were studied for demographics, asthma management, and headaches. Most of the patients were male, married, and university-educated. Sixty-one percent had uncontrolled asthma, and 47.3 percent of individuals had headaches, mostly migraines. Uncontrolled asthma was linked to greater headache prevalence. Gender, educational level, and headache type did not affect headache prevalence in demographic and asthma control subgroups. Co-occurring asthma and migraines may benefit from asthma control and treatment.

Conclusion: The research emphasizes the significant frequency of uncontrolled asthma and headaches among asthmatic patients. The association between asthma control and headache prevalence was statistically significant, highlighting the necessity for appropriate management and treatment techniques for both disorders. These findings have significant implications for health care providers and politicians seeking to improve the quality of life for those with asthma and co-occurring headaches.

## Introduction

Asthma is a chronic inflammatory condition caused by interactions between heterogenic genes and the environment that are yet poorly understood. It has bronchial hyperresponsiveness and variable airway blockage. Clinically, asthmatic patients experience recurrent attacks of coughing, wheezing, tightness in the chest, and difficulty breathing [1]. Anamnesis, a spirometry test, physiological findings, and pathological findings all contribute to the diagnosis of asthma [2]. Moreover, asthma is a common disease that causes comorbidity and mortality since Around 300 million people worldwide suffer from asthma which also contributes to 250,000 deaths annually [3]. In Saudi Arabia, there are limited studies on the prevalence of asthma. According to a study conducted in the Riyadh region, 11.3% of the study population was diagnosed with asthma by a physician [4].

In addition, headaches are a prevalent health issue that can be caused by neurological conditions as well as other long-term illnesses, and since asthma and migraine are co-occurring chronic diseases with episodic attacks that are considered to be caused by inflammatory and neurological processes, according to different studies, there are some beliefs that headaches are a common disease in asthma patients, especially migraine headache [5-7]. Patients with asthma commonly have migraine and tension-type headaches, and those who experienced headaches were mainly female. Also, the findings show a strong correlation between the reduced respiratory function test, allergies, and migraine-type headaches. In addition, compared to healthy controls, asthma patients were shown to have a higher prevalence of tension-type headache, a primary headache subtype [5]. Regarding a study conducted in California, only female patients with asthma were found to have an association between migraine-type headaches and asthma [8]. Moreover, patients receiving inhaled steroids had a decreased incidence of migraine-type headaches. The reason for this observation may be that steroids are a partial success in suppressing migraine-type headaches [5]. It is considered that substance P and platelet-activating factors play a role in the etiology of migraine-like headaches and asthma [9]. Additionally, it is recognized that certain medications such as (beta-blockers, salicylates, and non-steroid anti-inflammatory medicines) used to treat migraine-type headaches can also cause asthma [10,11]. In a study performed in Turkey, 60% of asthma patients were found to have a headache and 32.6% of them fulfilled the criteria for the migraine diagnosis. In their study, females were shown to have higher incidences of primary headache and migraine than males [7].

Regarding the characteristics and the type of headache among asthma patients, a study of 58 healthy control individuals and 93 asthmatic patients has shown only 19 of the control participants (32.8%) developed headaches, compared to 58 patients with asthma (62.4%). Seven patients (7.5%) experienced other types of headaches, whereas 32 patients (34.4%) had tension-type headaches, and 19 patients (20.3%) had migraine-type headaches. When compared to people without asthma who were in good health, asthma patients' frequency of headaches was considerably greater (p=0.001). Patients with asthma get migraine- and tension-type headaches more frequently than the general population. Asthma patients who have allergies and poor respiratory function test results have migraine-type headaches more frequently [5]. According to a study, episodic headache sufferers were much more physically disabled during headache bouts. On the other hand, patients who experienced a chronic daily headache reported a markedly higher incidence of emotional disorders. In contrast to patients with episodic headaches, those with persistent daily headaches had considerably worse mental health (P 0.05) [12]. Patients with persistent daily headaches appeared to have worse quality of life (QOL) during the interictal period than those with episodic headaches. Higher disability scores across all parts were observed in participants with chronic daily headaches [12]. The findings of this study demonstrate that headaches have a deleterious effect on a patient's QOL not just during attack phases but also during interictal intervals, and they highlight the significance of including this factor in future research investigating the effect of headache treatments [12]. Headaches could affect the QOL. In addition, asthma is also known to be a chronic disease that could affect the QOL for patients [13]. According to a study to assess the QOL among headache patients, it was found that the QOL was not only affected during the attack but patients could also be affected during the interictal period [12]. This magnified the need for increased awareness since asthma and headaches are both chronic diseases that could affect the life of the patients [6]. As mentioned before, many studies show a relationship between asthma and headaches, especially migraine and tension headaches. Studies also found that headache prevalence is more in asthma patients compared to the general population.

However, while we were searching about this topic, we noticed insufficient studies assessing the relationship between asthma and headache attacks in Saudi Arabia especially in the Qassim region. In our study, we assess the relationship between headaches and asthma in the Qassim region, Saudi Arabia. The aim is to determine the prevalence of headaches among asthma patients, the characteristics and type of headache, and the headache-related disability in asthma patients.

## Materials and methods

The study was conducted as an observational, cross-sectional study from 11 September 2022 to 14 May 2023, targeting asthmatic patients who were diagnosed and followed by a pulmonologist in the Qassim Region through a survey that was distributed through King Saud Hospital, King Fahad Specialist Hospital, Buraidah Central Hospital, and Qassim University Hospital. The sample size was calculated depending on the Roasoft calculator using a population size of 25,000, with hypothesized % frequency of outcome factor in the population (p): 50%+/-5, confidence interval of 95 % ending in a total sample of 379 participants. The sampling technique was random, with inclusion criteria of asthma patients who lived in the Al-Qassim region, Saudi Arabia, and were 16 years old or older, while exclusion criteria were patients who did not live in the Al-Qassim region and patients younger than 16 years old. 

The data were collected using a structured questionnaire consisting of three sections. The first section included sociodemographic factors. The second section included questions to assess the character of headaches in asthma patients using a valid web-based headache questionnaire developed by Kyung Min Kim, Wonwoo Lee, Kyoung Heo, and Min Kyung Chu from the Department of Neurology, Severance Hospital, Yonsei University College of Medicine [14]. The questionnaire consisted of seven items to diagnose migraine, probable migraine, and tension-type headache. The third section included specific questions about asthma control tests to assess the control of asthma between patients, in addition to questions about the family history of asthma and headaches. 

A pilot or preliminary study on 10% of the participants was done as a pre-test for our research instrument to ascertain any issue and barrier to recruit participants and to assess the acceptability of observation or interview protocol. The data from the pilot study were not included in the main study.

Data were analyzed using IBM SPSS Statistics for Windows, Version 24 (Released 2016; IBM Corp., Armonk, New York, United States), with qualitative data expressed as numbers and percentages and quantitative data expressed as mean and standard deviation. The chi-square (x2) test was used to assess the relationship between two or more qualitative variables. The Student t-test was used for comparing two quantitative normally distributed variables, and the ANOVA test was used for comparing more than two quantitative normally distributed variables with the significant level set at P-value <0.05. 

Ethical approval was sought from the Regional Ethical Committee of Qassim Region, KSA, and participants were ensured confidentiality and the freedom to withdraw from the study at any time. Informed consent was taken prior to filling out the questionnaire.

## Results

Table 1 presents the demographic factors of 528 asthmatic patients, including age, gender, marital status, and educational level. The mean age of the sample was 42.16 years, with a standard deviation of 14.74. The majority of the samples were male, accounting for 56.4%, while 43.6% were female. Regarding marital status, the majority were married, accounting for 66.9%, while single, widowed, and divorced participants accounted for 25.4%, 4.7%, and 3.0%, respectively. In terms of educational level, the majority had a university or higher education, accounting for 48.9%, while 28.4% had a secondary education, and 8.9% could read and write. Only 6.6% of the participants were illiterate.

**Table 1:** Demographic factors of the asthmatic patients (N=528)

		Count	Column N %
Age	Mean (SD)	42.16 (14.74)	
Gender	Male	298	56.4%
	Female	230	43.6%
Marital status	Single	134	25.4%
	Married	353	66.9%
	Widowed	25	4.7%
	Divorced	16	3.0%
Educational level	Illiterate	35	6.6%
	Read and write	47	8.9%
	Secondary education	150	28.4%
	University or higher	258	48.9%
	Higher education	38	7.2%

**Figure 1:**
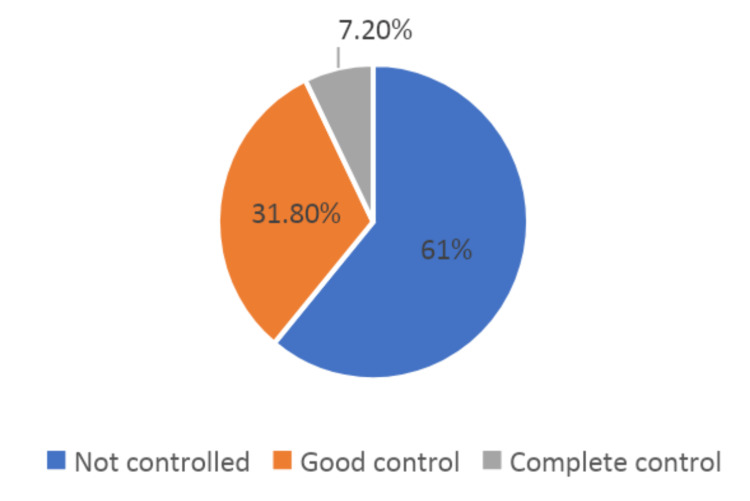
The asthma control distribution among patients

The data collected from 528 participants revealed that the majority of the asthmatic patients had uncontrolled asthma, as 61.0% of the participants reported not having their asthma under control. Only 31.8% of the participants reported having good control over their asthma, and a small proportion of the sample, 7.2%, reported complete control over their asthma (Figure 1).

Table 2 shows the distribution of asthma control among 528 participants based on gender, marital status, and educational level. The majority of both male and female participants reported uncontrolled asthma, with 61.4% and 60.4%, respectively. There was no significant difference in asthma control between genders (p-value=0.885). Among the marital status categories, single participants had the highest percentage of uncontrolled asthma (62.7%), followed by married participants (59.8%). However, there was no statistically significant difference in asthma control among different marital status categories (p-value=0.272). Regarding the educational level, illiterate participants had the highest percentage of uncontrolled asthma (68.6%), followed by those who could read and write (72.3%). However, there was no statistically significant difference in asthma control among different educational levels (p-value=0.267).

**Table 2:** The relation between asthma control and demographic factors of patients

		Asthma control						
		Not controlled		Good control		Complete control		P-value
		Count	Row N %	Count	Row N %	Count	Row N %	
Gender	Male	183	61.4%	95	31.9%	20	6.7%	0.885
	Female	139	60.4%	73	31.7%	18	7.8%	
Marital status	Single	84	62.7%	44	32.8%	6	4.5%	0.272
	Married	211	59.8%	110	31.2%	32	9.1%	
	Widowed	15	60.0%	10	40.0%	0	0.0%	
	Divorced	12	75.0%	4	25.0%	0	0.0%	
Educational level	Illiterate	24	68.6%	10	28.6%	1	2.9%	0.267
	Read and write	34	72.3%	11	23.4%	2	4.3%	
	Secondary education	83	55.3%	50	33.3%	17	11.3%	
	University or higher	158	61.2%	83	32.2%	17	6.6%	
	Higher education	23	60.5%	14	36.8%	1	2.6%	

Table 3 presents the prevalence of headaches among 528 asthmatic patients and their family members. Of the 528 participants, 47.3% reported experiencing headaches, while 52.7% reported no headaches. Among those who reported experiencing headaches, the majority had migraine-type headaches (52.4%), followed by probable migraine (24.0%), tension-type headaches (10.0%), and unclassified headaches (13.6%). Of the participants who experienced exacerbation attacks of asthma, 50.8% reported suffering from headaches after the attack. About 29.6% of the participants reported having a family member who suffers from a headache attack, while 70.4% reported no family history of headache. Among those who reported having a family member who suffers from a headache attack, 49.7% reported that the family member was not diagnosed with asthma, while 26.1% reported that the family member was diagnosed with asthma, and 24.1% did not know.

**Table 3:** The prevalence of headaches and type of headache among asthmatic patients

		Count	Column N %
Headache	No	278	52.7%
	Yes	250	47.3%
Type of headache	Migraine	131	52.4%
	Probable migraine	60	24.0%
	Tension-type headache	25	10.0%
	Unclassified headache	34	13.6%
Do you suffer from headache after the exacerbation attack of asthma?	No	123	49.2%
	Yes	127	50.8%
Is there someone in your family who suffers from a headache attack	No	176	70.4%
	Yes	74	29.6%
If your answer is yes, is the person who suffers from a headache diagnosed with asthma too?	No	99	49.7%
	Yes	52	26.1%
	I do not know	48	24.1%

Table 4 shows the distribution of headaches and type of headache among 528 asthmatic patients based on gender, marital status, educational level, and asthma control. Among the total samples, 47.3% reported experiencing headaches, with no significant difference in headache prevalence between males and females (p-value=0.434). Single participants had a higher prevalence of headaches (52.2%) compared to married participants (43.6%), with borderline statistical significance (p-value=0.050). There was no significant difference in the prevalence of headaches among different educational levels (p-value=0.813). Asthma control was significantly associated with headache prevalence (p-value=0.000), with higher prevalence of headaches reported among participants with uncontrolled asthma (55.6%) compared to those with good (38.1%) or complete control (18.4%). The majority of participants with headaches had migraine-type headaches (52.4%), while probable migraine and tension-type headaches accounted for 24.0% and 10.0%, respectively. There was no significant difference in the prevalence of different types of headaches among different demographic and asthma control subgroups. These findings suggest that asthma control is significantly associated with headache prevalence, and effective management and treatment strategies for asthma may also improve headache outcomes for individuals with co-occurring asthma and headaches.

**Table 4:** The distribution of headache and type of headache among 528 asthmatic patients based on gender, marital status, educational level, and asthma control

		Headache					Type of headache								
		No		Yes		P-value	Migraine		Probable migraine		Tension-type headache		Unclassified headache		P-value
		Count	Row N %	Count	Row N %		Count	Row N %	Count	Row N %	Count	Row N %	Count	Row N %	
Gender	Male	161	54.0%	137	46.0%	0.434	72	52.6%	31	22.6%	13	9.5%	21	15.3%	0.890
	Female	117	50.9%	113	49.1%		59	52.2%	29	25.7%	12	10.6%	13	11.5%	
Marital status	Single	64	47.8%	70	52.2%	0.050*	32	45.7%	21	30.0%	6	8.6%	11	15.7%	0.209
	Married	199	56.4%	154	43.6%		81	52.6%	34	22.1%	16	10.4%	23	14.9%	
	Widowed	10	40.0%	15	60.0%		13	86.7%	1	6.7%	1	6.7%	0	0.0%	
	Divorced	5	31.3%	11	68.8%		5	45.5%	4	36.4%	2	18.2%	0	0.0%	
Educational level	Illiterate	17	48.6%	18	51.4%	0.813	11	61.1%	4	22.2%	1	5.6%	2	11.1%	0.761
	Read and write	25	53.2%	22	46.8%		10	45.5%	9	40.9%	3	13.6%	0	0.0%	
	Secondary education	87	58.0%	63	42.0%		33	52.4%	12	19.0%	8	12.7%	10	15.9%	
	University	130	50.4%	128	49.6%		67	52.3%	31	24.2%	11	8.6%	19	14.8%	
	Higher education	19	50.0%	19	50.0%		10	52.6%	4	21.1%	2	10.5%	3	15.8%	
Asthma control	Not controlled	143	44.4%	179	55.6%	0.000*	97	54.2%	47	26.3%	14	7.8%	21	11.7%	0.318
	Good control	104	61.9%	64	38.1%		31	48.4%	12	18.8%	10	15.6%	11	17.2%	
	Complete control	31	81.6%	7	18.4%		3	42.9%	1	14.3%	1	14.3%	2	28.6%	

## Discussion

Patients who suffer from asthma frequently exhibit symptoms of an illness known as headache, which is known to impact patients' QOL. Numerous research studies looked into the connection between headaches and asthma and found that there was a connection. The frequency of asthma and chronic bronchitis was 1.5 times higher in patients with migraine and non-migraine headaches than in the normal population, and the presence of asthma and bronchitis was found to be associated with the frequency of headaches [15]. These findings are in accordance with the findings of the study known as "Head Hunt," which found that the frequency of headaches was associated with the presence of asthma and bronchitis. The current study studied the prevalence of headaches among asthmatic patients as well as the features of such headaches. According to the findings, almost half of the participants (47.3% of them) reported having headaches, and the majority of those who reported having headaches said they suffered from migraine-type headaches (52.4 percent). There is no consistent pattern across research regarding the prevalence of headaches among asthma patients. An earlier study carried out by Gungun et al. showed that the prevalence of headaches among asthmatic patients was observed to be 58 percent [5]. This is quite comparable to the prevalence that was found in the current study (47.3 percent). Additionally, Turan et al. observed that roughly sixty percent of their patients had headaches, indicating that headaches are quite common in asthma patients [7].

Several common biological variables, such as genetic predisposition, mast cell activation, platelet-activating factors, and poor arachidonic acid metabolism, have been reported to have a role in the pathogenesis of co-existing asthma and headaches. These factors include hereditary predisposition [9,16]. Several studies have revealed a connection between asthma, headaches of the migraine variety, and everyday headaches [9,15,17-19]. On the other hand, the diagnosis of asthma is not conclusive in the vast majority of these investigations; rather, it is exclusively reliant on the patient's medical history and the symptoms of asthma. Both the platelet-activating factor and the substance P substance have been hypothesized to play a role in the development of asthma as well as the production of migraine-type headaches [9]. In a different piece of research, only female patients were found to have an association between migraine-type headaches and asthma [8].

The majority of those who reported having headaches suffered from migraine-type headaches (52.4 percent). A probable migraine accounted for 24.0% of all headaches, a tension-type headache for 10%, and an unclassifiable headache for 13.6% of all headaches. Previous studies have shown that those who have asthma are more likely to experience migraine headaches than other types of headaches, which is consistent with these new findings [18]. Headaches of the migraine variety are distinguished by varying degrees of discomfort, ranging from mild to severe, and are frequently accompanied by nausea, vomiting, and sensitivity to light and sound [20]. Individuals who suffer from asthma may require a combination of pharmacological and non-pharmacological treatments in order to have effective management of headaches similar to migraines. In addition to asthma drugs like beta-agonists and leukotriene modifiers, which have been found to relieve migraine symptoms, non-pharmacological treatments including cognitive-behavioral therapy, stress reduction strategies, and lifestyle changes may also be beneficial in controlling both disorders [21].

In addition, the demographic characteristics that are connected with the occurrence of headaches among asthmatic patients were investigated in this study. When compared to married participants, the number of headaches reported by single participants was somewhat higher, but the difference did not reach statistical significance. On the other hand, there was no discernible variation in the frequency of headaches between the sexes or educational levels of the participants. These findings imply that the marital status of asthma patients may be a possible factor related to headache prevalence; however, additional studies are required to corroborate this finding. However, in another study, the authors found that both migraines and primary headaches are more common in girls than they are in males, according to the findings of this study (2.3 and 1.8 times higher, respectively) [7]. According to the findings of another study, the prevalence of migraines is approximately two to three times higher in women [22]. 

The current study suffers from a number of shortcomings, one of which is its cross-sectional design, which makes it impossible to determine whether or not there is a causal connection between asthma and headaches. In addition, the study relied on self-reported data, which may be prone to biases related to recall as well as social desirability bias. Despite this, the study reveals important insights into the prevalence of headaches among asthmatic patients as well as the characteristics of those headaches. These findings could help in the development of effective management and treatment strategies for people who simultaneously suffer from asthma and headaches.

## Conclusions

In conclusion, the present study provides insight into the significant occurrence of headaches among asthmatic patients, in particular among those whose asthma is not under control. According to the findings of the study, the presence of a spouse may potentially be a factor that contributes to the frequency of headaches experienced by asthmatic patients. These findings highlight the need for comprehensive management and treatment strategies for individuals who suffer from co-occurring asthma and headaches. Such techniques have the potential to improve these individuals' QOL as well as their overall health outcomes.
